# A comparative study on the effects of fungal and bacterial phytase with or without citric acid on growth performance, serum mineral profile, bone quality, and nutrient retention in broilers

**DOI:** 10.5455/javar.2024.k786

**Published:** 2024-06-09

**Authors:** Rakhi Chowdhury, Md. Aliar Rahman, Khan Md. Shaiful Islam, Mohammad Al-Mamun

**Affiliations:** Department of Animal Nutrition, Faculty of Animal Husbandry, Bangladesh Agricultural University, Mymensingh, Bangladesh.

**Keywords:** citric acid, non-phytate phosphorus, performance, phytase, retention

## Abstract

**Objective:**

Current research aimed to compare the effects of fungal and bacterial phytase with or without citric acid (CA) on growth performance, serum mineral profile, bone quality, and nutrient retention in birds given non-phytate phosphorus (nPP)-deficient diets.

**Materials and Methods:**

A total of 216 Indian River broiler chicks were disturbed into six groups, namely, i) positive control (PC), ii) negative control (NC) contained 0.2% lower nPP than that in the PC diet, iii) NC + fungal phytase (*Aspergillus niger*), iv) NC + fungal phytase with 2% CA, v) NC + bacterial phytase (*Escherichia coli*), and vi) NC + bacterial phytase with 2% CA.

**Results:**

Compared to the PC group, the NC group showed poor performance, serum phosphorus (P) content, P retention, and bone quality. However, with the inclusion of phytase, all these phenomena were improved. The addition of bacterial phytase showed better values compared with fungal phytase. The main effects of phytase were significant for the feed conversion ratio (FCR), metabolizable energy conversion ratio (MECR), and P retention. The addition of CA, either with fungal or bacterial phytases, did not show considerable beneficial effects on overall performances. However, the main effects of CA were significant on the FCR, MECR, and crude protein conversion ratio.

**Conclusion:**

Incorporating bacterial and fungal phytase into low-nPP diets enhanced the broiler’s performance. The effects of bacterial phytase were more apparent than those of fungal phytase. However, the efficacy of phytase based on the source might relate to dose, and other factors need further investigation.

## Introduction

Poultry diets usually contain phytate, the chief reserve form of phosphorus (P) in plant material, which restricts P bioavailability and creates ecological issues since excreted P pollutes the environment. Many studies have been carried out to solve this problem by reducing the amount of synthetic P and including the phytase enzyme in diets. The inclusion of phytase in the diet releases P from phytate, which can help compensate for dietary deficits of non-phytate P (nPP). Therefore, phytase was commercially launched in 1991, originated from *Aspergillus niger,* and is considered a first-generation phytase [1]. Thereafter, the new generation of phytase originating from *Escherichia coli* was found to be more effective in degrading the phytate molecules, allowing the release of a higher amount of P in the feed [2]. These two generations of phytase, fungal and bacterial, vary in their efficacy; in addition, their effect depends on some factors such as the amount of phytate in the diet, animal species, animal age, mineral intake through the diet, the origin of phytase, and the phytase dose in the diet [3]. Although many findings are available on the influence of most of the above-mentioned factors, very few articles focus on the influence of phytase sources and comparisons between the sources in terms of performance, nutrient utilization, and other characteristics in commercial broilers, so this area needs to be addressed.

It was reported that organic acid decreases the pH of the broilers, and adding it to diets increases the effectiveness of added phytase [4,5]. Additionally, the synergistic effect of dietary phytase with citric acid (CA) was informed by several scientists [6–8]. The mechanism can be explained by the fact that the intestinal pH of broilers may decrease with the addition of CA, which leads to an increase in the efficiency of phytase because it correlates with the concentration of other free cations and acidity. A concrete concept on the effect of phytase from different sources with or without CA is necessary. Thus, this research was intended to compare the effects of fungal (*A. niger*-derived) and bacterial (*E. coli*-derived) phytase enzymes with or without CA in broilers given low nPP diets.

## Materials and Methods

### Ethical approval

The experiment management, execution, and sample collection procedures were endorsed by the Animal Welfare and Experimentation Ethics Committee at Bangladesh Agricultural University, Mymensing (AWEEC/BAU/2022 (62)).

### Birds and housing

Two hundred and sixteen-day-old, mixed-sex commercial Indian River broiler chicks were purchased and reared for 35 days, considering the marketing age of broilers in Bangladesh. Chicks were weighed individually after arrival and then divided into six dietary groups, each containing three replications of twelve birds, all of which were balanced for body weight (BW). The brooder temperature was 32°C, whereas the room temperature was 29°C at the first week of brooding, which was done using electric bulbs. Throughout the experimental period, light was provided 24 h a day.

### Experimental diets

The corn-soybean meal-based diets (Table 1), namely the positive control (PC) diet, which was prepared following the guidelines of the NRC [9], and the negative control (NC) diet, which contained 0.2% lower nPP than the nPP level in the PC diet, the NC diet containing fungal phytase 500 FTU/kg feed (*A. niger* derived; Natuphos^®;^ E, BASF, Germany), the NC diet containing fungal phytase with 2% CA, the NC diet containing bacterial phytase 500 FTU/kg feed (*E. coli* derived; OptiPhos^®;^ Huvepharma, Bulgeria), the NC diet containing bacterial phytase with 2% CA. At the top of the ration, phytase and CA were added. Starter diets had 22.26% crude protein (CP) and 3,004 kcal of metabolizable energy (ME) per kg and were served from day one to day fourteen. Afterward, grower diets containing 20.95% CP and 3,114 kcal of ME per kg were given from 15 to 35 days of age.

**Table 1.** Ingredients and chemical composition of the experimental diets (starter phase: 1–14 days; grower phase: 15–35 days)^†^

Ingredients, %	Starter phase		Grower phase	
	PC	NC	PC	NC
Corn	42.70	43.20	47.20	47.50
Protein concentrate	15.00	15.00	11.00	11.00
Soybean meal	32.00	32.00	31.00	31.00
Limestone	0.50	1.25	0.70	1.50
Di-calcium phosphate	2.00	0.75	1.50	0.40
Soybean oil	7.00	7.00	7.80	7.80
Vit-min premix^††^	0.40	0.40	0.40	0.40
Methionine	0.10	0.10	0.10	0.10
NaCl	0.30	0.30	0.30	0.30

Analyzed composition, % (as fed basis)				
Crude protein	22.26	22.30	20.95	20.97
Crude fiber	4.80	4.81	4.64	4.13
Total P	0.78	0.60	0.69	0.50
Phytate P	0.33	0.35	0.31	0.32
Non-phytate P^¶^	0.45	0.25	0.38	0.18
Calcium	0.98	0.97	0.95	0.93
ME (kcal/kg)^ ¶^	3,002	3,006	3,109	3,120

^†^PC = positive control, diet formulated according to NRC [9] recommendation; NC = negative control, diet formulated according to NRC [9] recommendation except non-phytate P content, which was 0.2% lower than that in the PC diet; P = phosphorus; ME = metabolizable energy; Kcal = kilocalorie

^††^Each kg premix contained=Vitamin A palmitate, 6,600 IU; cholecalciferol, 2,200 IU; menadione dimethylpyridine bisulfite, 2.2 mg; riboflavin, 4.4 mg; pantothenic acid, 13 mg; niacin, 40 mg; choline chloride, 500 mg; biotin, 1 mg; vitamin B12, 22 μg; ethoxyquin,125 mg; iron, 50 mg; copper, 6 mg; zinc, 40 mg; manganese, 60 mg; selenium, 0.2 mg.

^¶^Non-phyatte P was calculated by subtracting the phytate P from total P.

^¶^Calculated nutrient content was based on ingredient composition data from the NRC [9].

### Experimental procedure

The birds were raised from day one to day twenty-eight in floor pens; after that, they were moved to wire cages and kept for 35 days. Birds had unlimited access to feed and water. The recording was taken on a daily and weekly basis for feed intake (FI) and BW, respectively. The ratio of FI to BW gain (gm feed/gm gain) was used to determine the feed conversion ratio (FCR). In contrast, the CP conversion ratio (CPCR) and ME conversion ratio (MECR) were determined by adopting a published procedure [10]. Excreta was collected between the ages of thirty-three and thirty-five and kept in a freezer at −20°C. Samples of excreta were collected carefully to prevent contamination with any external contaminants. Samples of frozen excreta were thawed, mixed, dehydrated, and powdered, and then samples were kept for further analysis of total P and nitrogen (N). At 36 days, live birds were slaughtered by neck-cut, and samples of blood were taken while it was bleeding [11,12] in a falcon tube and kept in an icebox to avoid blood clotting. The blood was then centrifuged using a centrifuge machine (Z 306, HERMLE, SN 76170124, HERMLE Labortechnik GmbH, Wehingen, Germany), and serum samples were extracted for the analysis of calcium, phosphorus, magnesium, and zinc in the prescribed protocol of a specific kit (Bio-vision) using a bio-analyzer (URIT-810, Chemistry Analyzer, SN 81080293, URIT Medical Electronic Co. Ltd., Guangxi, China). From each dressed bird, non-specific immunity-related organs such as the liver, spleen, thymus, and bursa were collected, weighed separately, and presented as the parentage of live weight. Shank and tibia bones were removed, and length and width were measured. Bone dry weight was determined following a 24-h drying at 100°C. Samples of bone were incinerated at 600°C for 24 h [13], and considering the bone dry weight, the ash percentage was measured. The weight/length index of the tibia and shank were also estimated following the described procedure [14].

### Chemical analyses and calculations

Following the standard procedure [15], samples of diets and excreta were examined for proximate components. The total P of samples was measured by following the ISO method [16], and the phytate P was measured by following the described procedure [17]. By subtracting the phytate P from the total P, nPP was calculated. Total P and N retention were calculated using a published equation [18]. The cost of used feed ingredients, chicks, and test materials (phytase and CA) were considered for the calculation of production costs.

### Statistical analyses

A one-way analysis of variance (ANOVA) was used to determine the statistical significance between the dietary groups at a significant level of 5% using SPSS (2011), and then Tukey’s multiple comparison test was performed. In tables, the data were shown as mean value ± standard deviation. A two-way ANOVA was further used to examine the main impacts of phytase source, CA inclusion, and the interactions between phytase source and CA inclusions in more detail (excluding the PC and NC groups).

## Results

### Growth performance

Data regarding the growth performances are shown in Table 2. Birds given the PC diet had a lower final BW (1,679 gm) than the specifically reported BW (2,231 gm) for Indian River broilers [19]. The weight declined by about 7% in the birds who consumed the NC diet in comparison to the final BW of the birds given the PC diet. However, the inclusion of phytase restored it, and further inclusion of CA boosted it numerically. The final BW’s trend and the BW gain were comparable. FI decreased numerically in the NC group; however, intake was the same across all other groups (*p* > 0.05). The PC group had an FCR value of 1.78, which was found to be worse than the comparable value of the as-hatched Indian River broiler (1.48; [19]), which was further worsened in the NC group (1.89). Interestingly, FCR was shown to be restored in phytase-included groups (1.82 and 1.79 in fungal and bacterial phytase-added groups, respectively), and further inclusion of CA improved the FCR (1.77) in both of the phytase-added groups. A similar pattern of results was noticed for CPCR and MECR.

**Table 2.** Effect of fungal and bacterial phytase with and without citric acid on performance parameters in broilers^†^.

Parameters	PC	NC	NC + fungal phytase^††^		NC + bacterial phytase ^††^		Source of variation (*p* value)		
			- CA	+ CA	- CA	+ CA	Phytase	CA	Phytase × CA
DOCW, (gm)	39.50 ± 1.15	40.67 ± 0.29	39.83 ± 0.95	40.58 ± 0.76	40.25 ± 0.75	40.83 ± 0.76	-	-	-
FBW (gm)	1679.25^a^ ± 38.62	1567.58^b^ ± 35.38	1681.83^a^ ± 34.12	1694.17^a^ ± 45.18	1695.08^a^ ± 37.75	1708.25^a^ ± 42.31	0.559	0.585	0.986
BWG (gm)	1639.75^a^ ± 39.44	1526.92^b^ ± 35.67	1642.00^a^ ± 34.92	1653.58^a^ ± 45.73	1654.83^a^ ± 37.10	1667.42^a^ ± 37.65	0.571	0.607	0.983
FI (gm)	2922.93 ± 63.11	2886.83 ± 50.51	2979.50 ± 53.95	2930.50 ± 78.00	2967.50 ± 71.97	2944.93 ± 59.78	0.976	0.379	0.740
FCR	1.78^c^ ± 0.009	1.89^a^ ± 0.014	1.82^b^ ± 0.018	1.77^c^ ± 0.003	1.79^bc^ ± 0.004	1.77^c^ ± 0.006	0.041	0.001	0.208
CPCR	0.34^c^ ± 0.002	0.36^a^ ± 0.003	0.35^bc^ ± 0.003	0.34^c^ ± 0.001	0.35^bc^ ± 0.001	0.34^c^ ± 0.001	0.051	0.001	0.257
MECR	4.95^c^ ± 0.024	5.25^a^ ± 0.040	5.04^b^ ± 0.050	4.92^c^ ± 0.007	4.98^bc^ ± 0.011	4.90^c^ ± 0.016	0.046	0.001	0.204
Survivability, %	100.00 ± 0.00	96.44 ± 4.81	97.22 ± 4.81	97.22 ± 4.81	97.22 ± 4.81	100.00 ± 0.00	0.580	0.580	0.580

^a-c^Means within a row not followed by common superscripts are different at* p *< 0.05.

^†^PC = positive control; NC = negative control; Fungal phytase = *A. niger, *derived (500 FTU/kg of feed); Bacterial phytase = *E. coli*, derived (500 FTU/kg of feed); CA = citric acid; DOCW = day old chick weight; FBW = final body weight; BWG = body weight gain; FI = feed intake; gm = gram; FCR = feed conversion ratio (gm feed/gm gain); CPCR = crude protein conversion ratio (gm protein/gm gain); MECR = metabolizable energy conversion ratio (MJ/gm gain).

^††^With and without the addition of 20 gm citric acid/kg feed.

According to the two-way ANOVA results, the main impacts of phytase and CA were statistically significant (*p* < 0.05) for FCR and MECR values but non-significant (*p* > 0.05) for final BW (FBW), BW gain (BWG), and FI. Although the main effect of CA was significant for CPCR, phytase did not show a similar result. Regarding all parameters related to broiler performance, there was no interaction effect.

### Serum mineral profile and non-specific immunity-related organ weight

Data regarding the parameters of serum mineral profile and non-specific immunity-related organ weight are presented in Table 3. The serum mineral profile data indicated that birds consumed the NC diet expressed significantly (*p* < 0.05) reduced serum P concentrations compared with the birds given the PC diet. Interestingly, the concentration increased (*p* < 0.05) when the phytase enzyme was supplemented with the NC diet. It is mentionable that both fungal and bacterial phytase-supplemented groups showed a higher (*p* < 0.05) concentration of serum P compared to the NC group. Nonetheless, a non-significant (*p* > 0.05) variation was noted between the fungal and bacterial phytase-supplemented groups. The concentration of serum P did not alter significantly (*p* > 0.05) when CA was included with phytase. Moreover, diets had no noticeable effect on serum calcium, magnesium, or zinc content. A similar response was observed in the cases of liver and bursa weight (%) in birds. The thymus and spleen weights of the NC group were significantly (*p < *0.05) lower than those of the other birds, and these weights were similar to those in the PC group since phytase was included. The results showed that the main impacts of phytase source and CA inclusions and their interactions were non-significant (*p* > 0.05) for serum mineral profile and non-specific immunity-related organ weight.

### Bone quality, nitrogen, and total phosphorus retention

Data on the bone quality parameters, nitrogen, and total P retention are presented in Table 4. The length or width of the tibia and shank bones were unaffected by the experimental diets (data not shown). In the NC group, every value in the tibia declined dramatically, and the dietary inclusion of phytase restored all the values. Further inclusion of CA with phytase just increased the values numerically. The highest values for tibia dry weight (5.81 gm), index (64.83 mg/mm), ash (43.58%), and P content (8.96%) were recorded in a group given the NC diet containing bacterial phytase with CA. There were only numerical differences between the fungal and bacterial phytase-included groups for the values of all those parameters, and the inclusion of CA either with fungal or bacterial phytase did not show any significant (*p* > 0.05) changes. Regarding the characteristics of the tibia and shank bones, the main effects of phytase and CA, as well as their interaction effects, were similar (*p* > 0.05).

Because there was 0.2% less P in the NC diet than the PC diet, birds given the NC diet showed a lower (*p* < 0.05) retention of P (44.43%) compared to the PC diet-fed birds (47.90%). Interestingly, a significant improvement in retention was observed after the addition of phytase to the NC diet. Notably, the retention of P in phytase and phytase with CA groups was considerably higher than that of the PC group. While the dietary CP content was essentially the same across all groups, nitrogen retention differed dramatically among them. The lower retention of nitrogen in birds from the NC group was recovered with the inclusion of phytase and became comparable with the PC group. The further inclusion of CA had no noticeable impact. The retention of P was noticeably (*p* < 0.05) impacted by the inclusion of phytase, whereas the main effect of CA was non-significant (*p* > 0.05) for either P or N retention. No interaction effect was observed either for N or P retention.

### Production cost

Data on production costs are presented in Table 5. Due to the lower FI per bird in the NC group and the lower percentage of dietary P, feed cost per bird was reduced in the NC diet-given group compared to the PC diet-given group. The inclusion of phytase and CA in the NC diet created a progressive increase in price per kg of feed, though FI was almost similar. The NC group exhibited the greatest production cost per kg BW, the lowest, but the same in both the PC group and the bacterial phytase-added groups. The addition of CA with fungal and bacterial phytase increased the production cost numerically and significantly (*p* < 0.05), respectively. The benefit-cost ratio (BCR) was calculated to be 1.108 and 1.050 in the PC and NC groups, respectively. The inclusion of fungal and bacterial phytase in the NC diet showed BCR values of 1.094 and 1.105, respectively, whereas the values were 1.079 and 1.082 in CA with fungal and bacterial phytase added to NC diets, respectively. The main impacts of phytase and CA were significant (*p* < 0.05) for the cost/kg BW of birds. No interaction was observed for production cost.

**Table 3.** Effect of fungal and bacterial phytase with and without citric acid on blood profiles and non-specific immunity-related organs in broilers^†^.

Parameters	PC	NC	NC + fungal phytase^††^		NC + bacterial phytase^††^		Source of variation (*p*-value)		
			- CA	+ CA	- CA	+ CA	Phytase	CA	Phytase × CA
**Serum minerals profile**									
Calcium (mg/dl)	9.51 ± 0.25	9.11 ± 0.15	9.37 ± 0.19	9.46 ± 0.24	9.38 ± 0.13	9.53 ± 0.27	0.762	0.349	0.782
Phosphorus (mg/dl)	6.13^a^ ± 0.08	4.45^c^ ± 0.15	5.59^b^ ± 0.17	5.78^ab^ ± 0.08	5.79^ab^ ± 0.22	5.77^ab^ ± 0.16	0.327	0.409	0.312
Magnesium (mg/dl)	1.75 ± 0.05	1.72 ± 0.04	1.77 ± 0.09	1.83 ± 0.08	1.81 ± 0.06	1.82 ± 0.06	0.752	0.355	0.531
Zinc (µg/dl)	189.26 ± 8.04	183.00 ± 6.26	185.19 ± 5.09	186.89 ± 3.45	191.42 ± 7.08	192.64 ± 6.39	0.105	0.668	0.943
**Non-specific immunity-related organs weight (%)**									
Liver	2.03 ± 0.05	1.98 ± 0.05	2.09 ± 0.06	2.03 ± 0.08	2.02 ± 0.08	2.01 ± 0.03	0.295	0.260	0.477
Spleen	0.12^a^ ± 0.01	0.09^b^ ± 0.01	0.11^a^ ± 0.01	0.12^a^ ± 0.01	0.12^a^ ± 0.01	0.13^a^ ± 0.01	0.076	0.076	0.256
Thymus	0.25^a^ ± 0.01	0.21^b^ ± 0.01	0.26^a^ ± 0.02	0.29^a^ ± 0.03	0.26^a^ ± 0.02	0.28^a^ ± 0.02	0.663	0.086	0.663
Bursa	0.19 ± 0.02	0.17 ± 0.01	0.20 ± 0.02	0.20 ± 0.02	0.20 ± 0.02	0.21 ± 0.01	0.710	0.710	1.000

^a-c^ Means within a row not followed by common superscripts are different at *p* < 0.05.

^†^PC = positive control; NC = negative control; Fungal phytase = *A. niger, *derived (500 FTU/kg of feed); Bacterial phytase = *E. coli*, derived (500 FTU/kg of feed); CA = citric acid; mg/dl = milligram per deciliter; µg/dl = microgram per deciliter; % = percent.

^††^With and without the addition of 20 gm citric acid/kg feed.

**Table 4.** Effect of fungal and bacterial phytase with and without citric acid on bone characteristics, nitrogen, and total phosphorous retention in broilers^†^.

Parameters	PC	NC	NC + fungal phytase^††^		NC + bacterial phytase^††^		Source of variation (*p*-value)		
			- CA	+ CA	- CA	+ CA	Phytase	CA	Phytase × CA
**Tibia**									
Dry wt. (gm)	5.63^a^ ± 0.15	4.99^b^ ± 0.15	5.48^a^ ± 0.25	5.70^a^ ± 0.19	5.67^a^ ± 0.12	5.81^a^ ± 0.06	0.368	0.234	0.632
Index (mg/mm)	60.84^a^ ± 2.93	55.05^b^ ± 1.18	61.61^a^ ± 2.28	62.33^a^ ± 1.04	62.25^a^ ± 1.35	64.83^a^ ± 1.74	0.051	0.056	0.370
Ash (%)	42.2^a^ ± 1.08	37.58^b^ ± 2.17	41.64^ab^ ± 1.53	42.31^a^ ± 1.88	42.29^a^ ± 1.82	43.58^a^ ± 1.41	0.664	0.652	0.829
Phosphorus (%)	8.90^a^ ± 0.09	7.68^b^ ± 0.10	8.78^a^ ± 0.08	8.88^a^ ± 0.10	8.87^a^ ± 0.16	8.96^a^ ± 0.14	0.423	0.399	0.652
**Shank**									
Dry wt. (g)	3.72^a^ ± 0.08	3.33^b^ ± 0.10	3.56^a^ ± 0.08	3.62^a^ ± 0.10	3.65^a^ ± 0.07	3.71^a^ ± 0.09	0.091	0.229	1.000
Index (mg/mm)	49.86^a^ ± 2.26	42.22^b^ ± 1.38	47.14^ab^ ± 2.50	49.01^a^ ± 1.91	48.70^a^ ± 3.02	52.79^a^ ± 1.59	0.082	0.057	0.433
Ash (%)	39.04^a^ ± 1.11	34.55^c^ ± 1.10	38.31^b^ ± 1.03	39.23^ab^ ± 0.89	39.63^ab^ ± 0.87	39.71^ab^ ± 0.68	0.114	0.352	0.434
Phosphorus (%)	7.93^a^ ± 0.21	6.99^b^ ± 0.10	7.76^a^ ± 0.08	7.82^a^ ± 0.06	7.83^a^ ± 0.07	7.89^a^ ± 0.07	0.170	0.127	0.814
**Nitrogen (N) and Phosphorous (P) retention (%)**									
N retention	64.21^a^ ± 0.40	60.37^b^ ± 1.55	63.79^a^ ± 0.61	64.45^a^ ± 0.54	64.37^a^ ± 0.45	65.04^a^ ± 0.61	0.106	0.072	0.992
P retention	47.90^c^ ± 1.05	44.43^d^ ± 0.58	52.25^b^ ± 0.57	53.18^b^ ± 0.53	54.45^a^ ± 0.59	54.71^a^ ± 0.60	0.001	0.109	0.340

^a-d^ Means within a row not followed by common superscripts are different at *p* < 0.05.

^†^PC = positive control; NC = negative control; Fungal phytase = *A. niger*, derived (500 FTU/kg of feed); Bacterial phytase = *E. coli*, derived (500 FTU/kg of feed); CA = citric acid; wt. = weight; Index = weight/length; gm = gram; mg/mm = milligram per milliliter; % = percent.

^††^With and without the addition of 20 gm citric acid/kg feed.

**Table 5.** Effect of fungal and bacterial phytase with and without citric acid on broiler’s cost analysis^†^ [in US dollars (USD)^ ††^].

Cost (35-d trial)	PC	NC	NC + fungal phytase^§^		NC + bacterial phytase^§^		Source of variation (*p*-value)		
			- CA	+ CA	- CA	+ CA	Phytase	CA	Phytase × CA
Cost (feed/bird) ^¶^	2.015^ab^ ± 0.044	1.975^b^ ± 0.035	2.047^ab^ ± 0.037	2.093^ab^ ± 0.056	2.041^ab^ ± 0.050	2.106^a^ ± 0.043	0.894	0.073	0.740
Cost (feed + chick)/bird	2.224^ab^ ± 0.044	2.185^b^ ± 0.035	2.257^ab^ ± 0.037	2.303^ab^ ± 0.056	2.251^ab^ ± 0.050	2.316^a^ ± 0.043	0.894	0.073	0.740
Cost (feed + chick)/kg BW	1.356^c^ ± 0.006	1.431^a^ ± 0.011	1.374^bc^ ± 0.007	1.393^b^ ± 0.005	1.360^c^ ± 0.001	1.389^b^ ± 0.006	0.015	<0.001	0.113
Benefit-cost ratio	1.108 ± 0.005	1.050 ± 0.008	1.094 ± 0.005	1.079 ± 0.004	1.105 ± 0.000	1.082 ± 0.004	0.014	<0.001	0.013

^a-c^ Means within a row not followed by common superscripts are different at *p *< 0.05.

^†^PC = positive control; NC = negative control; Fungal phytase = *A. niger*, derived (500 FTU/kg of feed); Bacterial phytase *= E. coli*, derived (500 FTU/kg of feed); CA = citric acid; BW = body weight; kg = kilogram.

^††^1 USD = 109.77 BDT.

^§^With and without the addition of 20 gm citric acid/kg feed.

^¶^Cost of test substances included.

## Discussion

Current research diets were prepared according to the NRC [9] nutrient recommendations, and these were almost similar to the “Indian River Nutrition Specification.” The overall lower BW of birds in the present research might be due to factors such as hand-mixed mash diets [20], the surrounding environment [21], and the slightly lower CP content (22.26%; analyzed value) during the initial phase. Diets with low nPP levels are linked to less effective growth [22], and maintaining the ideal Ca:nPP ratio improves broiler performance [23,24]. According to NRC [9] recommendations, approximately 2.2:1 and 2.5:1 Ca:nPP ratios were maintained in the PC diet during the starter and grower phases, respectively. However, the ratio became higher in the NC diet (3.8:1 in the starter phase and 5.1:1 in the grower phase) due to the reduction of the nPP level, leading to decreased FI numerically and BW gain significantly (*p* <0.05). Several researchers found similar patterns of response in birds supplied with Ca:nPP in higher ratios [25,26]. The detrimental effects of low dietary nPP levels were restored when either fungal or bacterial phytase were supplemented with diets, indicating that nPP generated from dietary phytate P through phytase activity helped birds make up the deficiency of P in the NC diet. There was an almost full restoration in the bacterial phytase-supplemented group, and the inclusion of CA further boosted the restoration non-significantly (*p* = 0.05). Several researchers have highlighted the beneficial impact of organic acids in broiler diets, whether they contain phytase or not [27,28]; however, reports of ineffectiveness have also been made [26,29].

Although the marginal addition of synthetic P to the NC diet reduced the cost per kg of feed, the depressed growth performance of birds receiving this diet resulted in a high production cost per kg of BW. Both the PC and bacterial phytase-added groups had similar production costs and the lowest production cost per kg BW. The cost-effectiveness of phytase and CA inclusion individually in broiler diets was stated by some researchers [30–33]. In this research, the cost of production was only raised by adding CA along with phytase without providing any significant beneficial effects on broiler performances.

The beneficial impact of phytase on growth performance in birds fed a low nPP diet allows for the prediction of higher P retention in the phytase-included groups, which is confirmed: due to the deficiency of nPP in the diet, the NC group retained less nPP than the PC group, but supplementation of phytase increased retention values. The body’s homeostatic process, which increases P retention and absorption at a low dietary intake relative to a normal level, may account for an increase in P retention at a low nPP diet [34]. The highest value (54.71%) of P retention was recorded in the bacterial phytase with CA-added groups. The value was notably (*p* < 0.05) greater than that in the fungal phytase-added groups with or without CA (52.25% and 53.18%). Compared with fungal phytase, P retention increased by around 4% in the bacterial phytase-added groups; further inclusion of CA increased the retention by nearly 1% more. In this instance, the source of phytase played a vital role. Compared to phytase from *A. niger*, phytase from *E. coli* has considerable relative activity at the stomach pH and stronger resistance to proteolytic degradation [34,35]. Even though every group’s diet was iso-nitrogenous, nitrogen retention tended to rise numerically in groups given bacterial phytase-added diets. Similar observations have been reported by researchers [36,37], who discussed that phytase works to reduce the portion of phytate-protein complexes that are resistant to being digested.

Reduction of dietary nPP leads to lower concentrations (mg/dl) of serum P, weight (gm), ash (%), and P (%) in the tibia, as well as shanks in broilers fed the NC diet in comparison with the broilers consumed the PC diet. The quantity of nPP in the current study is likely insufficient to permit the storage of P in the body of broilers, particularly in the bones, with adverse consequences for bird growth. However, supplementation of phytase with or without CA restored the negative effects and showed the values for the above-mentioned parameters are comparable with those of birds given the PC diet. The effect of the phytase source was not noticeable here; however, a higher percentage of tibia P was found in birds fed a bacterial phytase-added diet compared with a fungal one and a control.

Notably, the addition of CA, either with fungal or bacterial phytase, did not show any significant beneficial or detrimental impact on growth performance, serum mineral profiles, bone quality, or nutrient retention. The positive effect of CA combined with the phytase enzyme on broiler performance was reported by several researchers [5,7]; whereas some also observed negative or no effects on overall performances [26,38]. Optimum levels of these two compounds may represent a possible solution to improve P utilization and overall performance without any additive or synergistic effects in broilers.

## Conclusion

The inclusion of phytase in low nPP corn-soybean meal-based diets improved performance, bone quality, serum P concentration, and N and P retention in broilers. In most of these cases, the bacterial phytase-added group showed better results, though the difference with the fungal phytase-added group was non-significant, except for P retention. The inclusion of CA did not show appreciable effects, either with fungal or bacterial phytase. Therefore, it is difficult to comprehend the efficacy of phytase based on its source. Further investigation is necessary to clarify whether there is any relationship between dietary nPP level, phytase source, and dose.

## List of Abbreviations

*A. niger, Aspergillus niger; *ANOVA, analysis of variance; BW, body weight; CA, citric acid; CP, crude protein; CPCR, crude protein conversion ratio; *E. coli, Escherichia coli; *FCR, feed conversion ratio; FI, feed intake; gm, gram; ME, metabolizable energy, MECR, metabolizable energy conversion ratio; N, nitrogen; NC, negative control; nPP, non-phytate phosphorus; NRC, National Research Council; P, phosphorus; PC, positive control.
